# Predictors of outcomes in patients with mitral regurgitation undergoing percutaneous valve repair

**DOI:** 10.1038/s41598-020-74407-z

**Published:** 2020-10-13

**Authors:** Alberto Polimeni, Michele Albanese, Nadia Salerno, Iolanda Aquila, Jolanda Sabatino, Sabato Sorrentino, Isabella Leo, Michele Cacia, Vincenzo Signorile, Annalisa Mongiardo, Carmen Spaccarotella, Salvatore De Rosa, Ciro Indolfi

**Affiliations:** 1 Division of Cardiology, Department of Medical and Surgical Sciences, URT National Research Council (CNR), Magna Graecia” University, Viale Europa, 88100 Catanzaro, Italy; 2 Research Center for Cardiovascular Diseases, “Magna Graecia” University, Catanzaro, Italy; 3 Mediterranea Cardiocentro, Naples, Italy

**Keywords:** Interventional cardiology, Cardiac device therapy

## Abstract

Percutaneous mitral valve repair has been increasingly performed worldwide after approval. We sought to investigate predictors of clinical outcome in patients with mitral regurgitation undergoing percutaneous valve repair. The MITRA-UMG study, a single-centre registry, retrospectively collected consecutive patients with symptomatic moderate-to-severe or severe MR undergoing MitraClip therapy. The primary endpoint was the composite of cardiovascular death or rehospitalization for heart failure. Between March 2012 and July 2018, a total of 150 consecutive patients admitted to our institution were included. Procedural success was obtained in 95.3% of patients. The composite primary endpoint of cardiovascular death or rehospitalization for HF was met in 55 patients (37.9%) with cumulative incidences of 7.6%, 26.2%, at 30 days and 1-year, respectively. In the Cox multivariate model, NYHA functional class and left ventricular end-diastolic volume index (LVEDVi), independently increased the risk of the primary endpoint at long-term follow-up. At Kaplan–Meier analysis, a LVEDVi > 92 ml/m^2^ was associated with an increased incidence of the primary endpoint. In this study, patients presenting with dilated ventricles (LVEDVi > 92 ml/m^2^) and advanced heart failure symptoms (NYHA IV) at baseline carried the worst prognosis after percutaneous mitral valve repair.

## Introduction

Mitral valve regurgitation (MR) is the most common form of valvular heart disease and affects ~ 10% of individuals with an age > 75 years^1^. Recently, an analysis from a European registry showed that more than half of patients with severe symptomatic MR were refused by surgeons mostly for the high burden of comorbidities and impaired left ventricular function, highlighting the need for less invasive treatment options^2^. In the past ten years, several transcatheter techniques have been developed to treat valve heart diseases, including MR. The percutaneous mitral valve repair (PMVR) with MitraClip (Abbott, USA) system is based on the edge-to-edge technique that was first described by the surgeon Ottavio Alfieri. The feasibility and safety of MitraClip device to treat MR have been first evaluated in the EVEREST I trial^3^. Whether the randomized EVEREST II trial^4^ compared PMVR to Surgery in operable patients with a predominantly primary MR and showed higher safety and similar clinical results.

Therefore, PMVR was included as a treatment option in patients with severe MR at high risk for surgery (Class IIb recommendation) in both the ESC^5^ and AHA/ACC^6^ guidelines. Recently, the results of MITRA-FR and COAPT trials assessing the efficacy and safety of MitraClip in patients with severe secondary MR have been published^7,8^. The MITRA-FR trial shows no benefit of MitraClip with respect to medical therapy while COAPT was a strong positive trial in favor of the MitraClip intervention. Possible reasons for this sharp discordance include more selective patient recruitment in the COAPT trial compared to MITRA-FR trial, more severe MR and less dilated ventricles (LVEDVi 101 ml/m^2^ vs 135 ml/m^2^, respectively). However, large registries^9,10^ on MitraClip therapy demonstrated the continuous need for outcome data derived from industry-independent multicenter studies. In this study, a single-centre retrospective registry, we sought to evaluate the clinical outcomes and to identify predictors of rehospitalization for heart failure or cardiovascular death from a registry of patients with MR undergoing PMVR with the MitraClip system.

## Methods

### Study population

The MITRA-UMG (Percutaneous MITral valve RepAir at University Magna Graecia) observational single-centre retrospective study collected consecutive patients with symptomatic moderate-to-severe or severe MR undergoing MitraClip therapy between March 2012 to July 2018 at Magna Graecia University (Catanzaro, Italy). Inclusion criteria were: symptomatic MR 3 + /4 + in high-risk patients unsuitable for surgery after heart team consensus, suitable mitral valve anatomy according to the instruction for the use of the device, life-expectancy > 1-year. Exclusion criteria were: Patients already participating in other clinical studies or unwilling to complete follow-up visits for the duration of the study. Clinical, echocardiographic and procedural data were collected and reported into an electronic database. Surgical Risk was evaluated prospectively using the European System for Cardiac Operative Risk Evaluation (EuroSCORE II, https://www.euroscore.org/calc.html). Follow-up data were collected by qualified personnel from Magna Graecia University of Catanzaro using a standardized questionnaire as previously described^11^. The local ethics committee (Comitato Etico Azienda Ospedaliera “Mater Domini”) approved the study, and all patients provided written informed consent. The study conforms to the principles outlined in the Declaration of Helsinki. Importantly, the MITRA-UMG study is independent from industry.

### Definitions

Primary MR was defined as MR caused by a primary abnormality of one or more components of the valve apparatus (i.e., leaflets, chordae tendineae, papillary muscles, annulus). Secondary MR was defined as MR due to primary LV dysfunction with normal mitral valve leaflets and chords. The MR grade was assigned as recommended by the American Society of Echocardiography based on a validated integrative method^12,13^ and two expert observers (I.L., M.C.). Any disagreement was resolved by consensus. Acute procedural success was defined as successful implantation of one or more clip(s) with a post-procedure reduction of MR of 2 + or less at discharge.

### Study endpoints

The primary endpoint was the composite of cardiovascular death or rehospitalization for heart failure. The secondary endpoints were the single components of the primary endpoint and all-cause death, all-cause rehospitalization, severe bleedings. All endpoints were defined according to the Mitral Valve Academic Research Consortium (MVARC) criteria^14^.

### Statistical analysis

The normal distribution of continuous variables was explored with Kolmogorov–Smirnov test. Continuous variables following a normal distribution were presented as mean ± SD and compared using the unpaired-sample Student’s t-test. Otherwise, variables which didn’t follow a normal distribution were presented as median (interquartile range [IQR]) and were compared with the Mann–Whitney U test. Categorical data were presented as count (percentages) and compared with the chi-square test or Fisher exact tests. Kaplan–Meier analysis was used to derive the event rates and plot time-to-event curves. Univariate Cox regression analysis included all significant or probable risk factors. The variables with a P < 0.1 were introduced in a stepwise multivariable model and parameters with a p-value ≤ 0.05 were then considered statistically significant, as previously described^15^. Continuous variables were dichotomized according to the best ROC cut-off values calculated by Youden index method^16^. Statistical analyses were performed using the MedCalc Statistical Software version 14.8.1 (MedCalc Software, Ostend, Belgium).

## Results

### Patient population

We included a total of 150 consecutive patients, admitted to our institution between March 2012 and July 2018. Clinical, echocardiographic and procedural data are reported in Tables 1, 2.

**Table 1:** Baseline characteristics.

	All (n = 150)	Organic MR (n = 41)	Functional MR (n = 109)	P
Age, y ± SD	75 ± 6	78 ± 4	74 ± 6	0.004
Male, n (%)	96 (64)	20 (49)	76 (70)	0.017
BMI, kg/m^2^ ± SD	26.3 ± 4	25.7 ± 4	26.4 ± 4	0.333
EuroSCORE II, % ± SD	9.9 ± 10	5.9 ± 7	11.4 ± 11	0.003
Hypertension, n (%)	135 (90)	39 (95)	96 (88)	0.199
Diabetes, n (%)	50 (33)	7 (17)	43 (39)	0.009
Prior Revascularization, n (%)	70 (47)	3 (7)	67 (61)	< 0.001
Prior MI, n (%)	70 (47)	3 (7)	67 (61)	< 0.001
Prior valvular surgery, n (%)	8 (5)	3 (7)	5 (5)	0.507
Prior stroke/TIA, n (%)	16 (10)	3 (7)	13 (12)	0.415
Atrial fibrillation, n (%)	79 (53)	27 (66)	52 (47)	0.047
COPD, n (%)	35 (23)	7 (17)	28 (25)	0.266
CKD, n (%)	77 (51)	16 (39)	61 (56)	0.064
Creatinine, mg/dl ± SD	1.5 (2)	1,1 (0.3)	1.6 (2.4)	0.139
GFR, mL/min ± SD	52.4 (19)	53 (17)	52 (20)	0.731
NYHA class III, n (%)	99 (66)	17 (41)	82 (75)	< 0.001
NYHA class IV, n (%)	9 (6)	2 (5)	7 (6)	0.722

*BMI* body mass index, *MI* myocardial infarction, *TIA* transient ischemic attack, *COPD* chronic obstructive pulmonary disease, *CKD* chronic kidney disease; *GFR* glomerular filtration rate, *NYHA* new york heart association.

**Table 2:** Echocardiographic parameters.

	All (n = 150)	Organic MR (n = 41)	Functional MR (n = 109)	P
MR grade 4 + , n (%)	113 (75)	31 (76)	82 (75)	0.961
Effective regurgitant orifice area, mm^2^ ± SD	31 ± 11	27 ± 15	30 ± 11	0.229
Left ventricular diameters (end diastolic/end systolic), mm ± SD	59 ± 7 / 43 ± 10	54 ± 7/ 37 ± 9	60 ± 7 / 46 ± 9	< 0.001
Left ventricular end diastolic volume /BSA ml/m^2^ ± SD	96 ± 27	80 ± 24	103 ± 25	< 0.001
Left ventricular end diastolic volume /BSA > 92 ml/m^2^,n (%)	77 (51)	10 (24)	67(61)	< 0.001
Left ventricular ejection fraction, % ± SD	42 ± 11	55 ± 7,2	37 ± 8	< 0.001
Left atrium volume /BSA, ml/m^2^ ± SD	54 ± 17	60 ± 20	52 ± 16	0.065
sPAP, mmHg ± SD	45 ± 13	48 ± 15	44 ± 12	0.070

*MR* mitral regurgitation, *BSA* body surface area, *sPAP* systolic pulmonary artery pressure.

Procedural characteristics and In-hospital outcomes are reported in Table 3. Briefly, our population average age was 75 ± 6 years, of those 64% of patients were male. Overall, we registered a high burden of comorbidities including important cardiac (history of myocardial infarction [47%]) and non-cardiac (chronic kidney disease [CKD, 51%], chronic obstructive pulmonary disease [COPD, 23%], diabetes mellitus [33%] diseases. Mean EuroSCORE II was 9.9 ± 10. Most of the patients presented with severe HF (66% New York Heart Association (NYHA) class III and 6% in NYHA class IV, Table 1) and 51% with a left ventricular end-diastolic volume index > 92 ml/m^2^ (Table 2). A functional aetiology was classified in 73% of patients with a median left ventricular ejection fraction and left ventricular end-diastolic volume index of 37 ± 8% and 103 ± 25 ml/m^2^ respectively.

**Table 3:** Procedural characteristics and in-hospital outcomes.

	All (n = 150)	Organic MR (n = 41)	Functional MR (n = 109)	P
**Number of implanted clip**				
1, n (%)	77 (51)	22 (56)	55 (50)	0,726
2, n (%)	61 (40)	16 (40)	45 (41)	< 0.001
3, n (%)	1 (0.7)	1 (2)	0 (0)	0.102
4, n (%)	0 (0)	0 (0)	0 (0)	
Acute procedural success, n (%)	143 (95.3)	39 (95.1)	104 (95.4)	0.940
Total dose area product (DAP), Gy * cm^2^ ± SD	191 ± 123	176 ± 157	196 ± 107	0.515
Total fluoro time, min ± SD	38 ± 23	40 ± 29	38 ± 21	0.751
In-hospital death, n (%)	4 (3)	1 (2)	3 (3)	0.518
In-hospital stay, day (IQR)	8 (6–11)	7 (6–9)	8 (7–11.5)	0.017
In-hospital stroke, n (%)	1 (0.7)	0 (0)	1 (1)	0.994
In-hospital severe bleeding /transfusion, n (%)	7 (5)	2 (5)	5 (5)	0.940
In-hospital cardiac tamponade, n (%)	1 (0.7)	0 (0)	1 (1)	0.538
In-hospital partial clip detachment, n (%)	2 (1.3)	0 (0)	2 (2)	0.382
**MR at discharge**				
Grade I, n (%)	63 (42)	20 (49)	44 (40)	0.353
Grade II, n (%)	77 (51)	19 (46)	58 (53)	0.453
Grade III, n (%)	5 (3)	2 (5)	3 (3)	0.518
Grade IV, n (%)	1 (0.7)	0 (0)	1 (1)	0.538

*DAP* dose area product, *MR* mitral regurgitation.

### Procedural outcomes

Acute procedural success was achieved in 95.3% of patients. A mean number of 1.4 ± 0.6 MitraClips were implanted (total fluoroscopy time 38 ± 23 min; total DAP 191 ± 123 Gy * cm^2^) with no death recorded during the procedure. The median in-hospital stay was 8 days (IQR 6–11).

### In-hospital outcomes

The most common intra-hospital complication was severe bleeding or anaemia requiring blood transfusion (5%). In-hospital death was 3%. Other in-hospital complications were infrequent (< 2%) and mostly due to partial clip-detachment (1.3%).

### Clinical outcomes

The median follow-up was 585 days (IQR 372–981 days) with a complete 1-year follow-up in 145 of 150 (97%) patients.

The combined primary endpoint of cardiovascular death or rehospitalization for HF occurred in 55 patients (37.9%) with cumulative incidences of 7.6% at 30-days and 26.2% at 1-year, respectively.

At follow-up, a total of 25 patients (17%) died. The rates of all cause death within 30 days and between 30 days/1-year were 2.7% and 9.3% respectively. Only 7 patients (4.6%) died after 1-year. Rehospitalization for HF occurred in 49 patients (33%) with cumulative incidences of 5.3% and 21.3%, at 30 days and 1-year, respectively. Annualized rates for all the outcomes are reported in Table 4.

**Table 4:** One-year clinical outcomes.

Primary endpoint	1-year rate, n (%)
Rehospitalization for HF or CV death	38 (26.2)
**Secondary endpoints**	
All-cause mortality	18 (12.4)
Cardiac	14 (9,6)
Non-cardiac	4 (2.8)
All-cause rehospitalization	50 (33.3)
Heart failure	32 (21.3)
other	18 (12)
New-onset AF	2 (1.4)
Severe bleeding	4 (2.8)

*HF* heart failure, *CV* cardiovascular, *AF* atrial fibrillation.

### Predictors of cardiovascular death or rehospitalization for HF

Univariate and multivariate analyses are reported in Table 5. NYHA class IV (HR 19.48, 95% CI [6.01, 63.17], p < 0.0001) and LVEDVi > 92 ml/m^2^ (HR 3.63, 95% CI [1.45, 9.09], p = 0.0062), independently increased the risk of the primary endpoint at long-term follow-up.

**Table 5:** Predictors of CV Death or Rehospitalization for HF.

Univariate			Multivariate	
Variable	Hazard ratio [95% Confidence Interval]	P-value	Hazard ratio [95% Confidence Interval]	P-value
NYHA IV	3.64 [1.62, 8.15]	0.0018	19.48 [6.01, 63.17]	< 0.0001
LVEDVi > 92 ml/m^2^	2.57 [1.42, 4.63]	0.0019	3.63 [1.45, 9.09]	0.0062
LVEF < 42%	2.15 [1.20, 3.84]	0.01		
DAP > 303 Gy * cm^2^	2.29 [1.16, 4.51]	0.0173		
Euroscore II > 7%	2.32 [1.34, 4.02]	0.0028		

*NYHA* new york heart association, *LVEDVi* left ventricular end-diastolic volume index, *LVEF* left ventricular ejection fraction, *DAP* dose area product.

**Figure 1:**
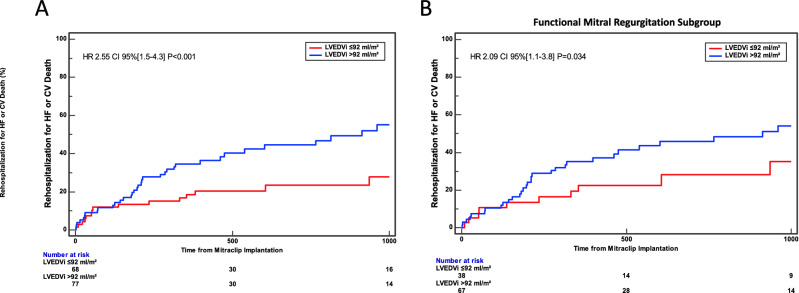
Primary endpoint of CV death or rehospitalization for HF according to left ventricular end-diastolic volume index. (**A**) At Kaplan–Meier analysis, a LVEDVi > 92 ml/m^2^ was associated with an increased incidence of the primary endpoint (HR 2.55, 95% CI [1.5, 4.3], p < 0.001). (**B**) Functional mitral regurgitation subgroup analysis. At Kaplan–Meier estimates, a LVEDVi > 92 ml/m^2^ was associated with an increased incidence of the primary endpoint (HR 2.09, 95% CI [1.1, 3.8], p = 0.034).

At Kaplan–Meier analysis, a LVEDVi > 92 ml/m^2^ was associated with an increased incidence of the primary endpoint of cardiovascular death or rehospitalization for heart failure (HR 2.55, 95% CI [1.5, 4.3], p < 0.001, Fig. 1A). Of note, a similar result was observed even in FMR subgroup (HR 2.09, 95% CI [1.1, 3.8], p = 0.034, Fig. 1B) (Supplementary file).

**Figure 2:**
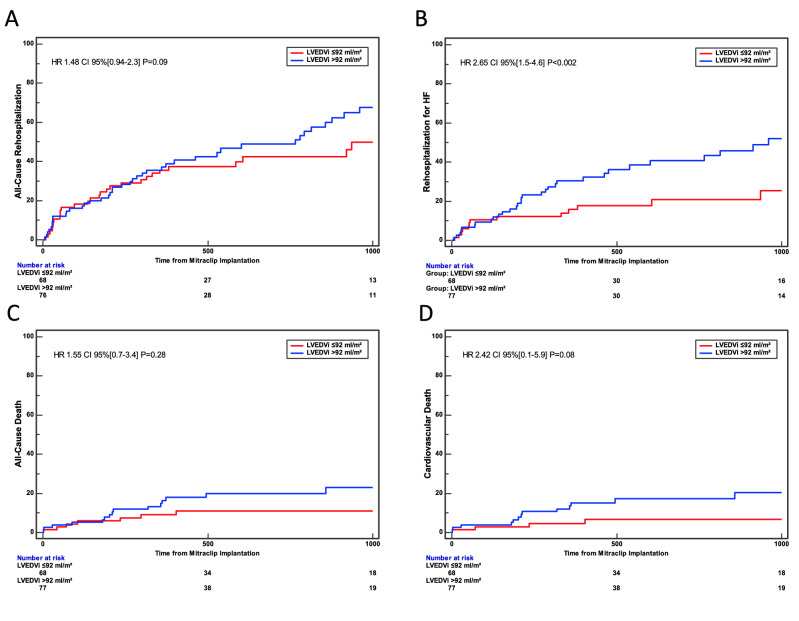
Secondary endpoints. (**A**) All-Cause Rehospitalization. At Kaplan–Meier analysis, no differences were found between the two groups (HR 1.48, 95% CI [0.94, 2.3], p = 0.09). (**B**) Rehospitalization for HF. At Kaplan–Meier analysis, a LVEDVi > 92 ml/m^2^ was associated with an increased incidence rehospitalization for HF (HR 2.65, 95% CI [1.5, 4.6], p < 0.002). (**C**) All-Cause Death. At Kaplan–Meier analysis, no differences were found between the two groups (HR 1.55, 95% CI [0.7, 3.4], p = 0.28). (**D**) Cardiovascular Death. At Kaplan–Meier analysis, no differences were found between the two groups (HR 2.42, 95% CI [0.1, 5.9], p = 0.08).

Interestingly, whether no differences were found for all-cause rehospitalizations between the two groups (HR 1.48, 95% CI [0.94, 2.3], p = 0.09, Fig. 2A), the risk for rehospitalization for heart failure after MitraClip implantation increased by 2.65-fold in patients with LVEDVi > 92 ml/m^2^ (95% CI 1.5–4.6, P < 0.002).

Finally, no differences were found between the groups for both all-cause death (HR 1.55, 95% CI [0.7, 3.4], p = 0.28, Fig. 2C) and cardiovascular death (HR 2.42, 95% CI [0.1, 5.9], p = 0.08, Fig. 2D).

## Discussion

The main findings of the present study are: (1) LVEDVi and NYHA class were independent predictors of Rehospitalization for HF or CV death in patients undergoing PMVR with the MitraClip system. (2) patients with a dilated left ventricle (i.e., LVEDVi > 92 mL/m^2^ based on cut-off calculated by ROC analysis) are more exposed to cardiovascular death or rehospitalization for heart failure.

Overall, these results underline the prognostic importance of effective MR reduction while ensuring the identification of a good number of patients with considerably decompensated ventricles and/or advanced NYHA class who may benefit less from PMVR.

The cumulative incidence of 1-year all-cause death in our study was 12.4%. This finding closely relates to data recently published in a meta-analysis of 16 studies^17^, in a large multicentre registry^18^ and in several single-centre studies^19–21^.

The reduction of rehospitalization for HF is a fundamental goal of PMVR therapy. In our registry, 1- year HF-related rehospitalizations after MitraClip implantation occurred in 21.3% of the patients. This finding is in line with Transcatheter Valve Treatment Sentinel Pilot Registry (TCVT)^18^ which reported a 1-year Kaplan–Meier incidence of 22.8%.

A robust predictive value of LV dimension for the functional outcome of patients has also been reported in surgical studies^22–24^. Recently, Zimarino and colleagues^25^ performed a meta-analysis of 2 RCTs and 7 non-randomized observational studies. They reported by means of a meta-regression analysis that larger left ventricular end-diastolic volume index (LVEDVi) is closely related to a higher risk of all-cause mortality, CV mortality and cardiac-related hospitalization after PMVR. Similarly, the GIOTTO registry showed favorable acute and 30-day safety and efficacy^9^. Interestingly, we expanded these findings identifying a cut-off of 92 ml/m^2^ of LVEDVi, which has proven to be independently correlated with CV death or rehospitalization for HF. Therefore, our data suggested that larger baseline LVEDVi might undermine the estimated advantage of PMVR, while patients with smaller LVEDVi achieved a better clinical outcome.

Previous trials and registries have assessed the prognostic impact of NYHA functional class^26,27^. A recently conducted multicentre study involving more than 800 patients from the German Mitral Valve Registry showed a lower survival rate at short-term follow-up in patients with baseline NYHA class IV in comparison to those on other functional classes^26^. Likewise, Capodanno et al.^28^ found NYHA class IV to predict rehospitalization after MitraClip implantation. Similarly, in our cohort, we identified NYHA class IV at baseline as an independent predictor of CV death and rehospitalization at follow-up.

However, these findings are far from being conclusive, and further studies are needed to address these issues.

## Limitations

The present study has some limitations. First, it was retrospective in design, with no control group and was conducted at a single-centre. Secondly, the sample size, mostly the primary MR group, and the number of events was relatively small. Lastly, further data, such as 6-MWT and QoL data, were not available in MITRA-UMG study.

## Conclusions

In searching the ideal phenotype of patients who benefit most of percutaneous mitral valve repair, those presenting with dilated ventricles (LVEDVi > 92 ml/m^2^) and advanced heart failure symptoms (NYHA IV) at baseline carried the worst prognosis at long-term.

## Supplementary information

Supplementary file1

## Data Availability

Anonymized data are available for external analysis from the corresponding author upon request.
