# Supplementary material for: Predictors of outcomes in patients with mitral regurgitation undergoing percutaneous valve repair
Source: Sci Rep. 2020 Oct 13;10:17144. doi: 10.1038/s41598-020-74407-z (PMC7555528; doi:10.1038/s41598-020-74407-z)
Supplement: Supplementary file 1 — Supplementary file1 [file 41598_2020_74407_MOESM1_ESM.pdf]

Supplementary data

Predictors of Outcomes in Patients with Mitral Regurgitation Undergoing Percutaneous Valve Repair

Alberto Polimeni, MD[1,2], Michele Albanese, MD[1], Nadia Salerno, MD[1], Iolanda Aquila, MD[1], Jolanda Sabatino, MD,[1,2], Sabato Sorrentino, MD,[1,2], Isabella Leo, MD[1], Michele Cacia, MD[1], Vincenzo Signorile, MD[1], Annalisa Mongiardo, MD[1], Carmen Spaccarotella, MD[1], Salvatore De Rosa, MD[1,2], Ciro Indolfi, MD[1,2,3].

[1]Division of Cardiology, Department of Medical and Surgical Sciences, "Magna Graecia" University, Catanzaro, Italy

[2]Research Center for Cardiovascular Diseases, "Magna Graecia" University, Catanzaro, Italy

[3] Mediterranea Cardiocentro, Naples, Italy

Running title: Predictors of outcome after Mitraclip

Corresponding author:
Ciro Indolfi, MD
Chief, Department of Medical and Surgical Sciences
and Director, URT National Research Council (CNR)
Magna Graecia University
Viale Europa
Catanzaro 88100, Italy
e-mail: indolfi@unicz.it
Phone: +3909613647668
fax: +3909613647153

Supplementary Figure 1

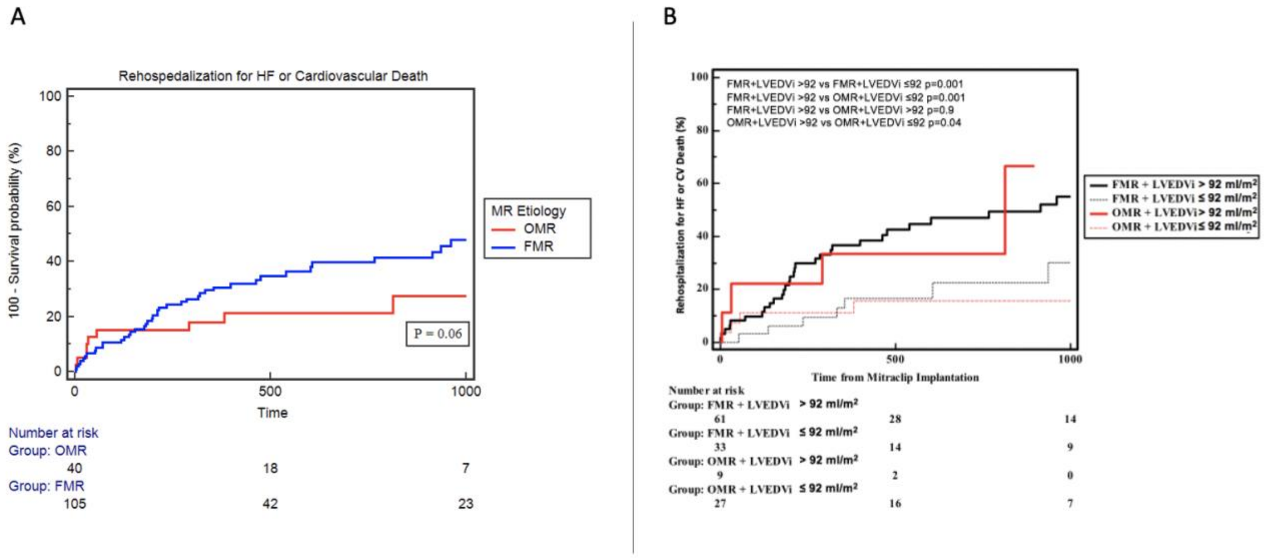

Supplementary Figure 1. Primary Endpoint of CV Death or Rehospitalization for HF according to MR Etiology

A) No differences were found between FMR and OMR groups for the primary endpoint.

B) Primary Endpoint of CV Death or Rehospitalization for HF according to MR Etiology and Left Ventricular End-Diastolic Volume index. At Kaplan-Meier analysis, a LVEDVi >92 ml/m2 was associated with an increased incidence of the primary endpoint regardless of the MR aetiology (functional or organic).
